# Pan-genome analysis of *Streptococcus suis* serotype 2 highlights genes associated with virulence and antibiotic resistance

**DOI:** 10.3389/fmicb.2024.1362316

**Published:** 2024-02-21

**Authors:** You Zhou, Teng Tu, Xueping Yao, Yan Luo, Zexiao Yang, Meishen Ren, Ge Zhang, Yuanyuan Yu, Aiping Lu, Yin Wang

**Affiliations:** ^1^Key Laboratory of Animal Diseases and Human Health of Sichuan Province, College of Veterinary Medicine, Sichuan Agricultural University, Chengdu, China; ^2^Law Sau Fai Institute for Advancing Translational Medicine in Bone and Joint Diseases (TMBJ), School of Chinese Medicine, Hong Kong Baptist University, Kowloon Tong, Hong Kong SAR, China; ^3^Guangdong-Hong Kong-Macao Greater Bay Area International Research Platform for Aptamer-based Translational Medicine and Drug Discovery (HKAP), Kowloon Tong, Hong Kong SAR, China; ^4^Institute of Integrated Bioinformedicine and Translational Science (IBTS), School of Chinese Medicine, Hong Kong Baptist University, Kowloon Tong, Hong Kong SAR, China

**Keywords:** SS2, pan-genome, GWAS, the core genome, the accessory genome, virulence, antibiotic resistance

## Abstract

*Streptococcus suis* serotype 2 (SS2) is a Gram-positive bacterium. It is a common and significant pathogen in pigs and a common cause of zoonotic meningitis in humans. It can lead to sepsis, endocarditis, arthritis, and pneumonia. If not diagnosed and treated promptly, it has a high mortality rate. The pan-genome of SS2 is open, and with an increasing number of genes, the core genome and accessory genome may exhibit more pronounced differences. Due to the diversity of SS2, the genes related to its virulence and resistance are still unclear. In this study, a strain of SS2 was isolated from a pig farm in Sichuan Province, China, and subjected to whole-genome sequencing and characterization. Subsequently, we conducted a Pan-Genome-Wide Association Study (Pan-GWAS) on 230 strains of SS2. Our analysis indicates that the core genome is composed of 1,458 genes related to the basic life processes of the bacterium. The accessory genome, consisting of 4,337 genes, is highly variable and a major contributor to the genetic diversity of SS2. Furthermore, we identified important virulence and resistance genes in SS2 through pan-GWAS. The virulence genes of SS2 are mainly associated with bacterial adhesion. In addition, resistance genes in the core genome may confer natural resistance of SS2 to fluoroquinolone and glycopeptide antibiotics. This study lays the foundation for further research on the virulence and resistance of SS2, providing potential new drug and vaccine targets against SS2.

## 1 Introduction

*Streptococcus suis* is a gram-positive coccus with a capsule. In previous studies, *Streptococcus suis* is divided into 35 serotypes (type 1–34, type 1/2), according to the different capsular polysaccharide (CPS) antigens (Fittipaldi et al., 2012). However, recent studies indicated that certain serotypes (type 20, 22, 26, and 32–34) are not associated with *Streptococcus suis* (Hill et al., 2005; Nishibori et al., 2013). Among them, *Streptococcus suis* serotype 2 (SS2) is the most common and virulent. SS2 primarily infects through wounds, causing acute septicemia, meningitis, arthritis, endocarditis, pneumonia, and other diseases in pigs (Zhu et al., 2006). It can also infect humans, leading to bacterial meningitis or toxic shock-like syndrome (Tang et al., 2006; Jiang et al., 2020).

With the advancement of sequencing technologies, an increasing number of species' genome sequences are being uploaded (Pareek et al., 2011). Particularly, the hybrid assembly of third-generation sequencing data and second-generation sequencing data is currently the mainstream approach for completing bacterial genome drafts (Loman and Pallen, 2015). Whole-genome sequences contribute to deepening our understanding of individual organisms (Orsini et al., 2016). Unfortunately, a single genome cannot reflect how genetic variations drive the pathogenic mechanisms within bacterial species (Tettelin et al., 2005; Maturana and Cárdenas, 2021). With the introduction of the pan-genome concept, the genetic variation trends and scope within bacterial populations can be described (Medini et al., 2005). The pan-genome consists of the core genome present in all strains and the accessory genome present in one or more strains (Vernikos, 2020). Moreover, with the increase in the number of genomes, core genomes and accessory genomes may exhibit differences in functionality, metabolic pathways, and resistance (Rasko et al., 2008; Mira et al., 2010). Currently, there have been reports of an open pan-genome composed of 19 SS2 strains, showing notable differences in pili and prophage regions (Guo et al., 2021). However, due to the limited number of samples, the specific differences in the core genome and accessory genome remain unknown.

The development of SS2 disease typically begins with the colonization of bacteria in the upper respiratory tract (Xia et al., 2019). During this process, various virulence factors of SS2 contribute to initial adhesion, immune evasion, and host invasion (Ji et al., 2016; Li et al., 2017). For example, CPS is the most representative virulence factor of *Streptococcus suis* (Zheng et al., 2013). It not only forms the basis for serotyping but also protects the bacteria from phagocytosis (Xia et al., 2019). Although several virulence factors have been proven to play a role in the early stages of infection, there are still quite a few virulence factors that remain unclear.

Currently, the most effective approach for treating *Streptococcus suis* involves antibiotics, as there are no commercially available vaccines (Palmieri et al., 2011). However, with the overuse of antibiotics in farming, the increasing prevalence of multidrug-resistant strains is limiting the options for effective antibiotics. Studies have reported a slow increase in resistance of *Streptococcus suis* to tetracyclines and macrolide/lincosamide antibiotics, which are widely used in the global animal sector (Dechêne-Tempier et al., 2023). Hence, it is necessary to explore relevant resistance genes to investigate the types of resistance within the SS2 population.

In this study, a strain of *Streptococcus suis* serotype 2 was isolated from a pig farm in Sichuan Province, China, and the complete genome sequence of the strain was obtained. Subsequently, we reported a whole-genome association study on the genomic sequences of 230 naturally isolated strains of SS2. This research unveiled significant distinctions between the core genome and accessory genome of SS2, such as in the transport and metabolism of essential substances, resistance to external conditions, and genetic reproduction. Additionally, our findings revealed virulence genes associated with the SS2 infection process and suggested its potential intrinsic antibiotic resistance.

## 2 Materials and methods

### 2.1 Diseased pig and bacteria isolation

The samples of diseased pig were collected from a pig farm located in Sichuan, China. All diseased pigs exhibited symptoms of anorexia, tremors, and joint swelling. The nasal secretions, joint fluid, and blood from diseased pigs were individually streaked onto trypticase soy agar (TSA) containing 5% fetal bovine serum and cultured at a constant temperature of 37°C for 18 h. Subsequently, grayish-white, semi-transparent, and smoothly surfaced circular colonies were selected for Gram staining and identification of isolated strains through 16S rRNA gene sequencing (Lane, 1991).

### 2.2 DNA extraction

In this study, the Bacterial DNA Kit (OMEGA, Norcross, USA) was used to extract bacterial DNA. Due to the direct correlation between Nanopore sequencing quality and input DNA quality, unnecessary centrifugation and shaking should be avoided during the DNA extraction process to minimize DNA fragmentation. The DNA solutions were quantified using a Nanodrop 2000 and a Qubit 3.0 Fluorometer (Thermo Fisher Scientific, CA, USA).

### 2.3 Library preparation and sequencing

The Ligation Sequencing Kit (SQK-LSK109) was used to construct Nanopore sequencing libraries according to the manufacturer's instructions, and MinION (Oxford Nanopore, Cambridge, UK) was used for sequencing. Subsequently, Illumina libraries were constructed using a Nextera XT Kit (Illumina, San Diego, CA, USA) followed by 150 bp paired-end sequencing on either the NextSeq 550 platform (Illumina, San Diego, CA, USA).

### 2.4 Base-calling and data processing

Firstly, guppy (V4.0.11, https://community.nanoporetech.com, accessed on 5 June 2023) was used for base-calling with a high accuracy model. Secondly, NanoFilt (V2.8.0, https://anaconda.org/bioconda/nanofilt, accessed on 5 June 2023) was used to filter the raw data, with a filtering threshold set at a *Q*-value >10 and a minimum read length of 1,000 bp, aiming to select a dataset with higher quality. Finally, the Illumina sequencing data were subjected to quality control via fastp (V0.23.3, https://github.com/OpenGene/fastp, accessed on 5 June 2023), followed by filtering to remove adapters and low-quality reads.

### 2.5 Genome assembly and integrity assessment

Firstly, Flye (V2.9.1, https://github.com/fenderglass/Flye, accessed on 10 September 2023) were employed for the initial assembly using filtered Nanopore sequencing data. Secondly, Pilon (V1.23, https://github.com/broadinstitute/pilon, accessed on 10 September 2023) was used for error correction of the bacterial genome via Illumina sequencing data. Thirdly, Bandage (V0.9.0, https://github.com/rrwick/Bandage, accessed on 10 September 2023) was used to check whether the contig formed a circular structure. Subsequently, Quast (V5.2.0, https://github.com/ablab/quast, accessed on 10 September 2023) was evaluated the corrected and uncorrected genome sequences. Finally, Busco (V5.4.7, https://busco.ezlab.org/, accessed on 12 september 2023) was used to assess the assembled bacterial genome.

### 2.6 Minimal inhibitory concentration (MIC)

It was carried out according to the standard microdilution method recommended by the Clinical and Laboratory Standards Institute (CLSI; Shryock, 2002; CLSI, 2018; Feßler et al., 2023). Seven types of commonly used SS2 drugs were selected, namely macrolides, lincomycins, β-lactams, cephalosporins, tetracyclines, glycopeptides and fluoroquinolones. *Streptococcus pneumoniae* ATCC 49619 was used as a quality control strain for drug susceptibility.

### 2.7 Information of bacterial strains

In this study, the complete genomes of 230 SS2 strains were retrieved to construct pangenome. These strains were available in March 2023 from NCBI (ftp://ftp.ncbi.nih.gov/genomes/all/). In addition, all strains were subjected to Busco assessment, with completeness exceeding 95%, to exclude genomes of low quality. Information about the 230 strains is summarized in Supplementary Table 1.

### 2.8 Pan-genome construction

To sustain the consistency and reliability of gene prediction and annotation, the Prokaryotic Genome Annotation System (Prokka) pipeline (V1.14.5 https://github.com/tseemann/prokka, accessed on 5 September 2023) was uniformly applied to all the 230 SS2 genomes. Based on the GFF3 files produced by Prokka, the Roary program (V3.13.0, https://github.com/sanger-pathogens/Roary, accessed on 6 October 2023) was used to construct the pan-genome with a minimum percentage identity of 95% between each predicted protein homolog. The decision not to choose 100% of the strains was to avoid misclassification caused by low-quality genomes or genome defects in individual strains, ensuring that true core genes were not overlooked during the annotation and classification process.

### 2.9 Gene annotation tool

In order to annotate the core genome and accessory genome, a variety of annotation tools and databases were utilized, as indicated in the Table 1.

**Table 1:** Gene annotation tools and databases.

**Name**	**URL**	**Purpose**
eggNOG-mapper	https://github.com/eggnogdb/eggnog-mapper (Cantalapiedra et al., 2021)	COG annotation
Interproscan	https://ftp.ebi.ac.uk/pub/software/unix/iprscan/ (Jones et al., 2014)	GO annotation
KEGG	https://www.genome.jp/kegg/ (Kanehisa and Goto, 2000)	KEGG annotation
VFDB	http://www.mgc.ac.cn/VFs/ (Liu et al., 2022)	Virulence factor annotation
CAZy	http://www.cazy.org/ (Drula et al., 2022)	Carbohydrate enzyme annotation
PHI	http://www.phi-base.org/ (Urban et al., 2020)	PHI annotation
CARD	https://card.mcmaster.ca (Alcock et al., 2023)	Drug resistance gene annotation

## 3 Result

### 3.1 Genome assembly and quality assessment

**Figure 1:**
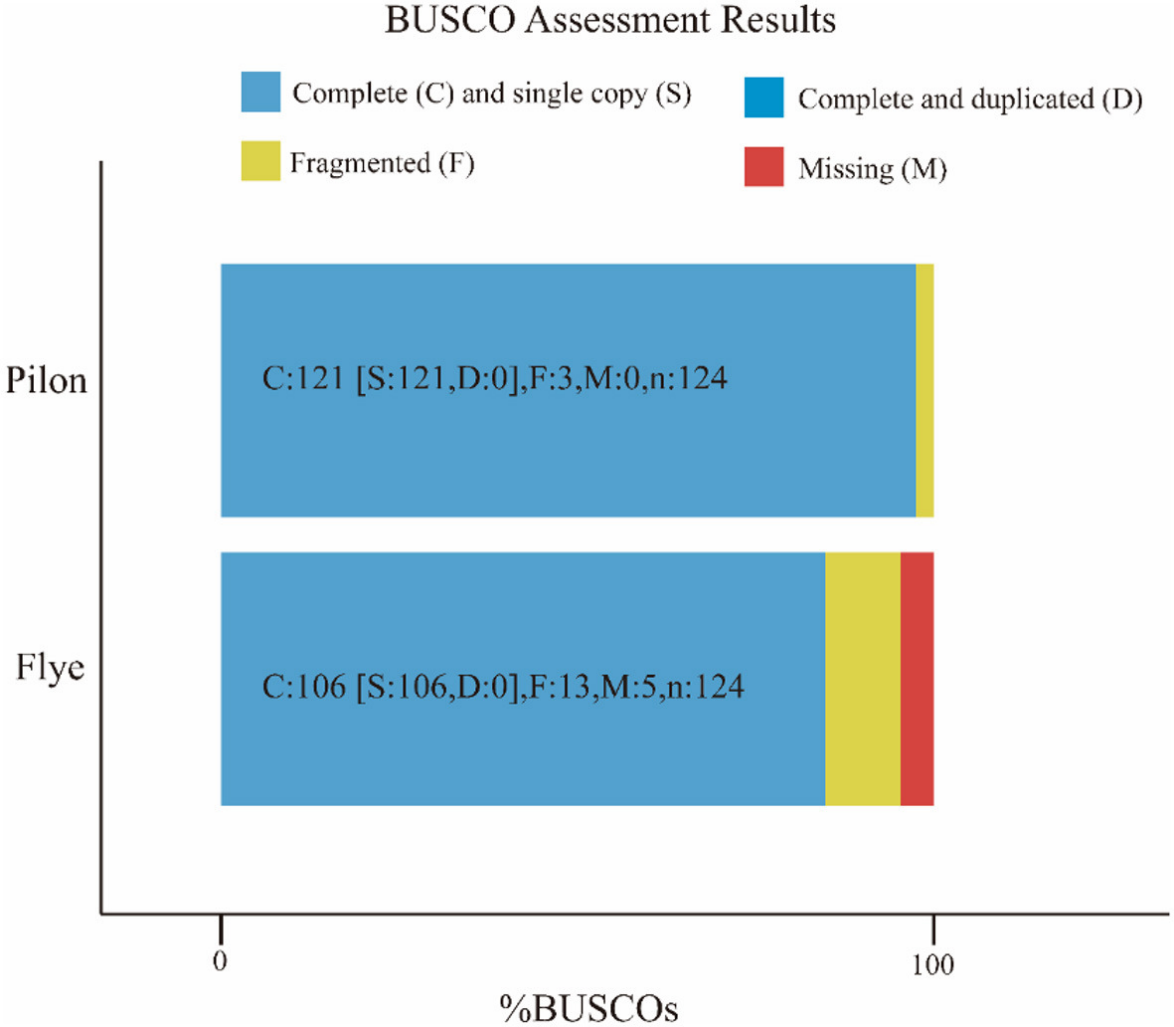
Busco visualization results. Compare the genome completeness after introducing second-generation sequencing data. It is evident that, in contrast to the initial bacterial genome assembly by Flye, the genome corrected by Pilon appears more comprehensive and contiguous, devoid of any missing segments. The specific differences are shown in the Table 1.

The attempt to directly assemble the bacterial genome using the filtered data with Flye resulted in a single contig of length 2,093,244 bp, closely matching the average size of the SS2 genome. This outcome suggested that the DNA extraction quality was good. After filtering, it was possible to assemble the bacterial genome directly. However, due to single-base errors in Nanopore sequencing, Illumina sequencing data will be used for correction in subsequent analyses. Illumina sequencing generated a total of 4.2 GB raw data, including 5,863,541 reads with a length of 151 bp and a GC content of 42%. The corrected and uncorrected genome sequences were evaluated using Quast, and the results are shown in Table 2. Pilon mainly corrected small indels and GC content since a contig forming a circular structure was already assembled in Flye. The final total length of the contig was 2,096,025 bp, with a GC content of 41.96%. Busco was employed for the integrity assessment of the genome sequences before and after correction, as shown in Figure 1. The genome sequence directly assembled using Flye exhibited 13 fragments and five missing genes. After correction with Illumina sequencing data, the integrity of the obtained genome was further improved. The number of genome fragments was reduced, and the missing genes were corrected. Therefore, the genome sequence corrected by Pilon is considered the final complete genome sequence of SS2 obtained in this study.

**Table 2:** Quast assessment results.

**Class**	**Software**	**Length (bp)**	**GC (%)**	**Contig**	**N50**
Preliminary results	Flye	2,094,582	41.23	1	2,094,582
Results after correction	Pilon	2,096,025	41.96	1	2,096,025

### 3.2 Genome composition analysis

**Figure 2:**
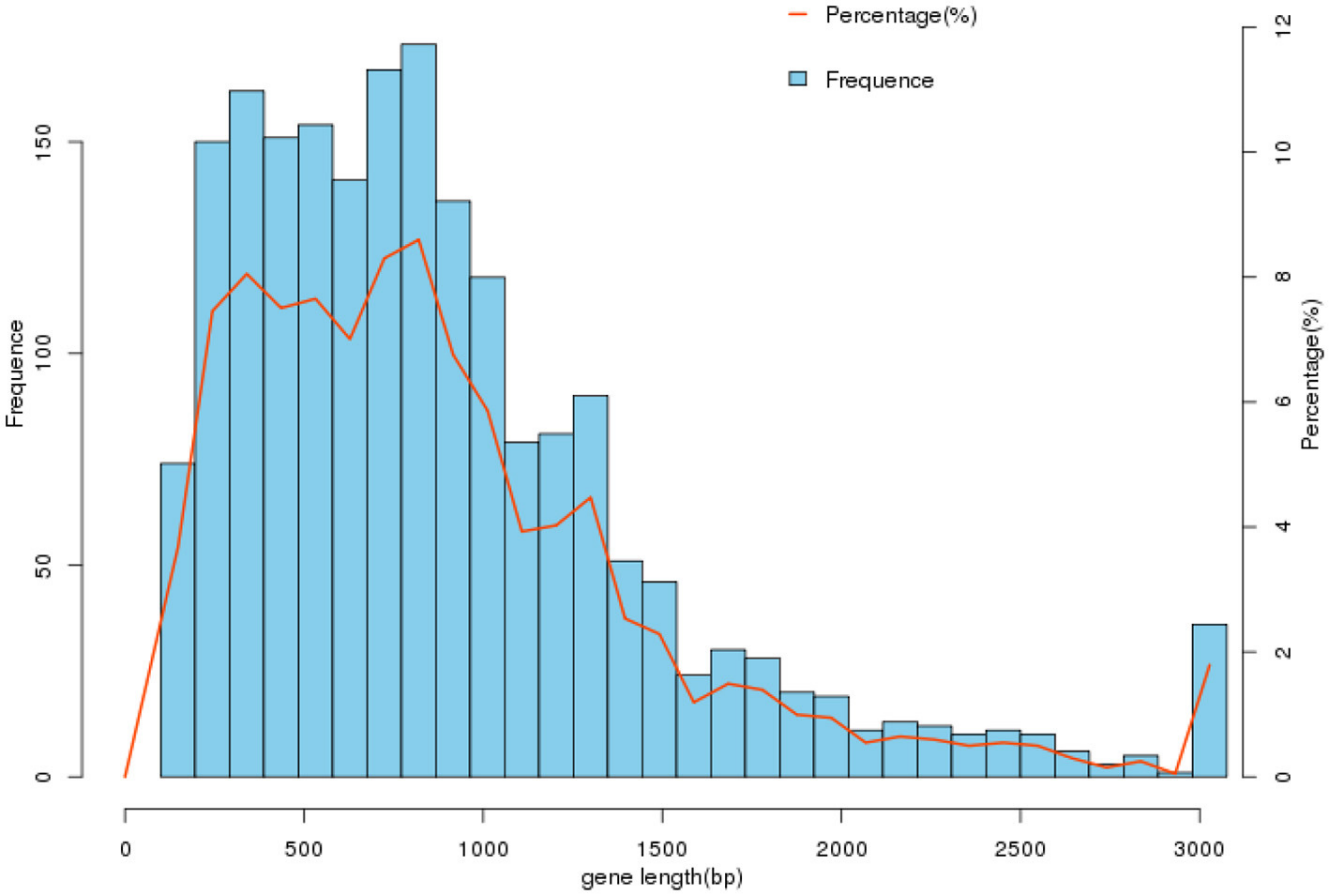
Gene length distribution. In the gene set cnzyss2-311, the gene lengths are predominantly distributed within the range of 0–3,000 bp, with the highest number of genes falling between 500 and 1,000 bp. The specific differences are shown in the Table 2.

Using Prodigal (V2.6.3, https://github.com/hyattpd/Prodigal, accessed on 10 September 2023) for gene prediction resulted in 2019 genes of varying lengths with a GC content of 41.96%, as shown in Table 3 and Figure 2. Genome composition analysis revealed 57 tRNA, 12 rRNA (including 4 5S_rRNA, 4 16S_rRNA, and 4 23S_rRNA), and nine sRNA. Additionally, 25 scattered repetitive sequences (16 short scattered repeats, seven long scattered repeats, and 2 DNA elements) and 46 tandem repeat sequences were identified. The genome also contained two CRISPR sequences, 4 GIs (Genomic Islands), and one prophage sequence. The relevant data has been uploaded to NCBI with the Bioproject ID PRJNA1041968, named cnzyss2-311.

**Table 3:** Genetic statistics.

**Counts**	**Total length**	**Maximum length**	**Minimum length**	**GC content**
2,019	1,837,305	6,309	90	41.96%

### 3.3 Antimicrobial susceptibility profiles

Using the broth microdilution method, the minimum inhibitory concentration (MIC) of isolated strains of *Streptococcus suis* type 2 was determined. The results were interpreted according to the CLSI standard (Table 4). The research results indicated that the isolated SS2 strain in this experiment exhibits high resistance to various antibiotics of different classes, including glycopeptides, tetracyclines, β-lactams, cephalosporins, and macrolides, reaching multidrug resistance. Additionally, it showed varying sensitivity to fluoroquinolones, including susceptibility to ofloxacin (S), intermediate resistance to levofloxacin (I), and resistance to trovafloxacin (R).

**Table 4:** Determination of minimum inhibitory concentration (MIC) results for cnzyss2-311 strain using microdilution broth method.

**Antibiotic category**		**MIC (μg/mL)**	**Susceptible (S)**	**Intermediate (I)**	**Resistant (R)**
Macrolides	Erythromycin	8	≤ 0.25	0.5	≥1
Cephalosporins	Ceftazidime	2	≤ 0.5	-	-
	Cefepime	32	≤ 0.5	-	-
β-lactams	Penicillin	32	≤ 0.12	-	-
	Penicillin G sodium salt	16	≤ 0.06	0.12–1	≥2
	Amoxicillin	64	≤ 0.5	1	≥2
Tetracyclines	Doxycycline	4	≤ 0.5	1	≥2
	Tetracycline	16	≤ 2	4	≥8
Lincomycins	Lincomycin	64	-	-	≥2
Glycopeptides	Vancomycin	2	≤ 1	-	-
Fluoroquinolones	Ofloxacin	0.25	≤ 2	4	≥8
	Levofloxacin	4	≤ 2	4	≥8
	Trovafloxacin	4	≤ 1	2	≥4

### 3.4 Construction and phylogenetic analysis of the SS2 pan-genome

**Figure 3:**
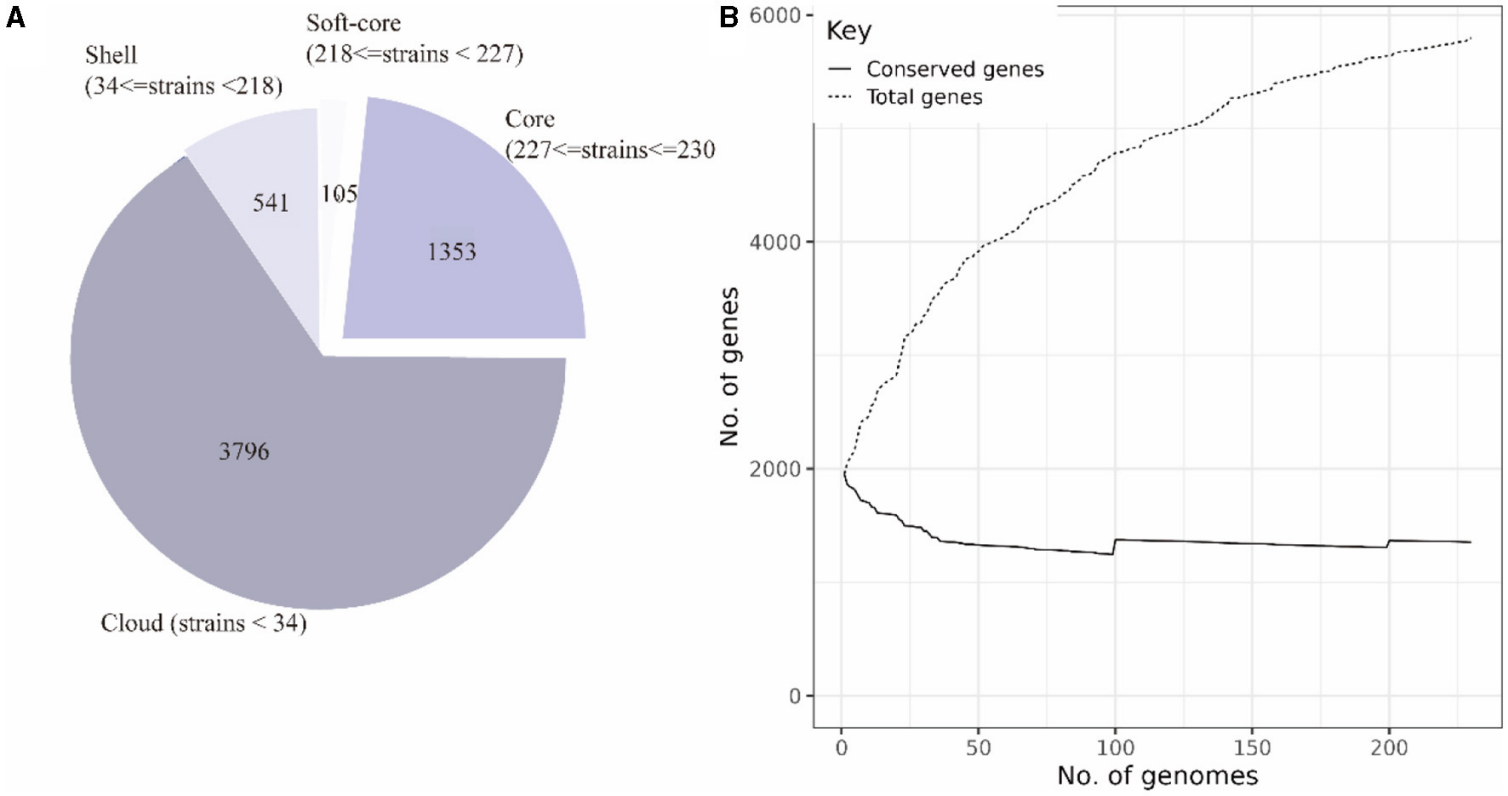
Composition of the SS2 pan-genome **(A)**. The pan-genome comprises 5,795 genes, with the core genome consisting of 1,458 genes and the accessory genome containing 4,337 genes. Openness of the pan-genome **(B)**. As the total number of genes increases, the count of core genes in the pan-genome gradually decreases.

The pan-genome of 230 SS2 strains was constructed after uniform annotation of their genomic sequences. The results indicated that the SS2 pan-genome comprises a total of 5,792 genes. Among them, there were 1,353 core genes shared by several strains ranging from 99 to 100%. In addition, there are 105 soft-core genes carried by 95–99% of strains; 541 shell genes carried by 15–95% of strains; and 3,796 cloud genes shared by 15% of strains (Figure 3A). However, considering that the functions of the four gene sets were not fully understood, only the core genome and accessory genome were used in the following analysis.

According to the composition of the SS2 pan-genome, it was found that the core genome of SS2 consists of 1,458 genes, accounting for 25.12% of the pan-genome, while the accessory genome was composed of 4,337 genes, indicating a high degree of genome variability in SS2. As shown in Figure 3B, the pan-genome was open, allowing continuous acquisition of foreign genes to adapt to different environments.

### 3.5 Gene function annotation

**Figure 4:**
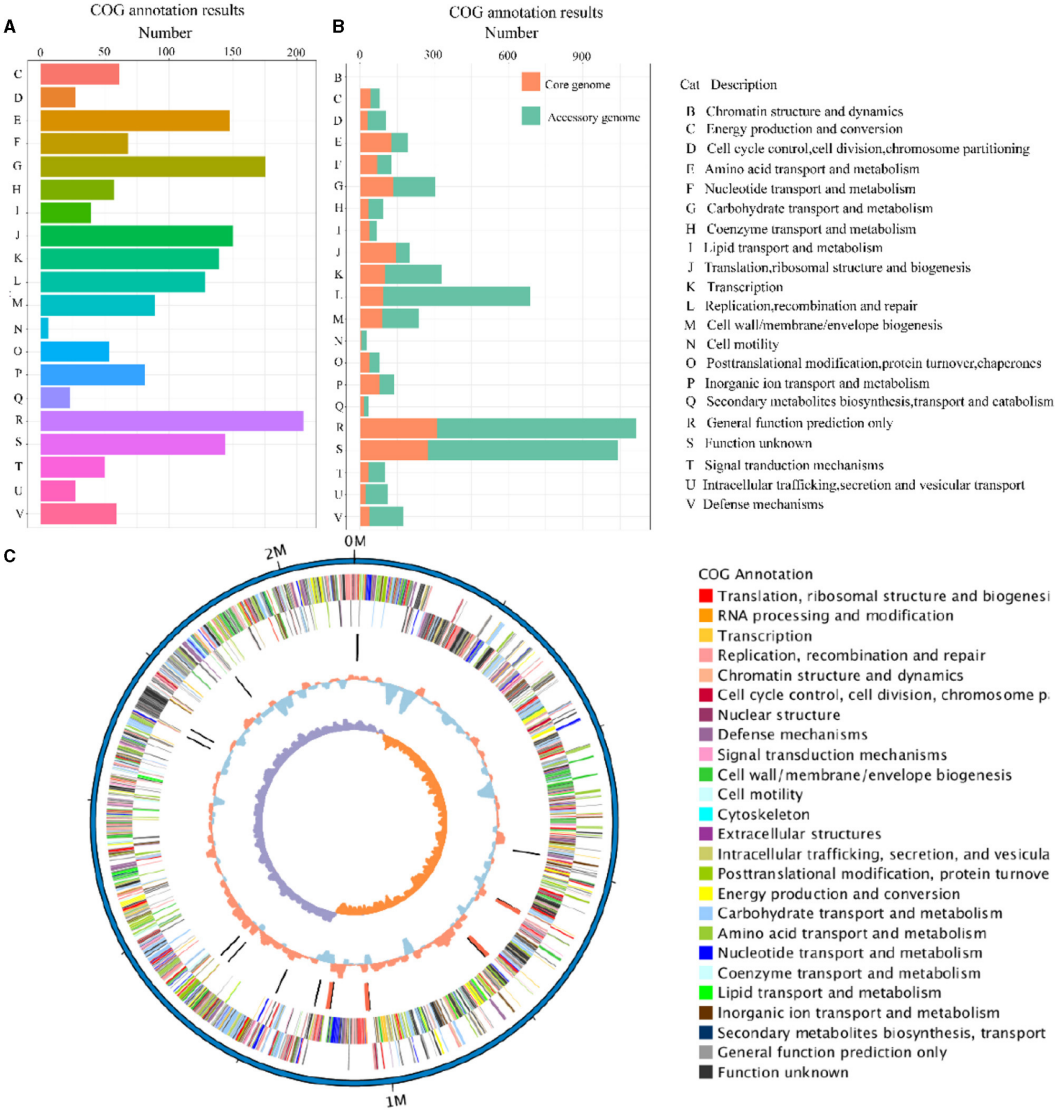
The COG annotation results of the cnzyss2-311 strain **(A)**. Different colors represent distinct COG annotation categories. The COG annotation results of the pan-genome **(B)**. The core genome and accessory genome are distinguished using orange and green, respectively. In addition, all COG annotation categories are labeled with letters, as shown on the right side of the figure. Circos diagram **(C)**. The result is shown that the outermost circle represents the genomic sequence coordinates. Moving inward, the circles represent: forward strand genes (color-coded by COG classification), reverse strand genes (color-coded by COG classification), non-coding RNA (black for tRNA, red for rRNA), GC content (red indicates above the mean, blue indicates below the mean), and GC skew (GC skewness, measuring the relative abundance of G and C, used to mark the starting and ending points in circular chromosomes).

The eggNOG-mapper (V5.0, https://github.com/eggnogdb/eggnog-mapper, accessed on 10 September 2023) was employed for functional annotation by aligning with the Cluster of Orthologous Groups of proteins (COG) database to analyze and infer gene functions. The genes obtained in Section 3.2 were annotated through COG analysis and the specific results are shown in Figure 4A. The functions of the genes in the SS2 strain obtained in this study are mainly concentrated in categories such as E (Amino Acid Transport and Metabolism), G (Carbohydrate Transport and Metabolism), J (Translation, Ribosomal Structure, and Biogenesis), K (Transcription), L (Replication, Recombination, and Repair), R (General Function Prediction Only), and other functions directly related to bacterial survival. Moreover, ~140 genes still have unknown functions. In addition, for the assembled genome sequence, combined with the predicted results of coding genes, a circular genome map was drawn to comprehensively display the features of the genome, and the result is shown in Figure 4C.

The COG annotation results for the pan-genome are shown in Figure 4B and Table 5. The results indicated that the core genome participated in various aspects of bacterial life processes. Moreover, almost all functions included a certain number of core genomes. The main functions of the core genome were concentrated in COG categories such as C (energy production and conversion), E (amino acid transport and metabolism), F (nucleotide transport and metabolism), J (translation, ribosomal structure, and biogenesis), P (inorganic ion transport and metabolism), which were biologically significant for maintaining bacterial metabolism.

**Table 5:** Cluster of Orthologous Groups of Proteins (COG) annotation of core genome and accessory genome.

**Class**	**Description**	**Core genome**	**Accessory genome**
B	Chromatin structure and dynamics	NA	1
C	Energy production and conversion	42	38
D	Cell cycle control, cell division, and chromosome partitioning	29	75
E	Amino acid transport and metabolism	124	68
F	Nucleotide transport and metabolism	69	59
G	Carbohydrate transport and metabolism	133	169
H	Coenzyme transport and metabolism	33	60
I	Lipid transport and metabolism	37	29
J	Translation, ribosomal structure, and biogenesis	145	53
K	Transcription	98	230
L	Replication, recombination and repair	93	593
M	Cell wall/membrane/ envelope biogenesis	88	148
N	Cell motility	6	19
O	Posttranslational modification, protein turnover, and chaperones	37	41
P	Inorganic ion transport and metabolism	77	61
Q	Secondary metabolites biosynthesis, transport, and catabolism	15	18
R	General function prediction only	312	805
S	Function unknown	275	767
T	Signal transduction mechanisms	33	66
U	Intracellular trafficking, secretion, and vesicular transport	21	89
V	Defense mechanisms	37	137

The accessory genome was annotated to all functional categories. It was annotated to categories not involved in the core genome (B, chromatin structure and dynamics). Additionally, COG annotation of the accessory genome was mainly concentrated in D (cell cycle control, cell division, and chromosome partitioning), L (replication, recombination, and repair), G (carbohydrate transport and metabolism), H (coenzyme transport and metabolism), indicating that the accessory genome was not only involved in bacterial division and reproduction but also involved the transport metabolism of necessary substances, showing a certain degree of essentiality. On the one hand, the accessory genome in K (transcription), O (post-translational modification), and U (intracellular trafficking, secretion, and vesicular transport) annotated quantities were significantly higher than the core genome, which might reveal the crucial role of the accessory genome in bacterial protein expression and transport processes. On the other hand, the accessory genome was concentrated in M (cell wall/membrane/envelope biogenesis), T (signal transduction mechanisms), and V (defense mechanisms). These three categories were related to the perception, response, defense, and adaptation of bacteria to changes in the external environment, maintaining survival and reproduction. Moreover, it might provide selective advantages and enrich population diversity. However, whether in the core genome or accessory genome, a large number of genes were still annotated to categories of unknown function (S), requiring further research.

### 3.6 The GO annotation of the core genome and the accessory genome

**Figure 5:**
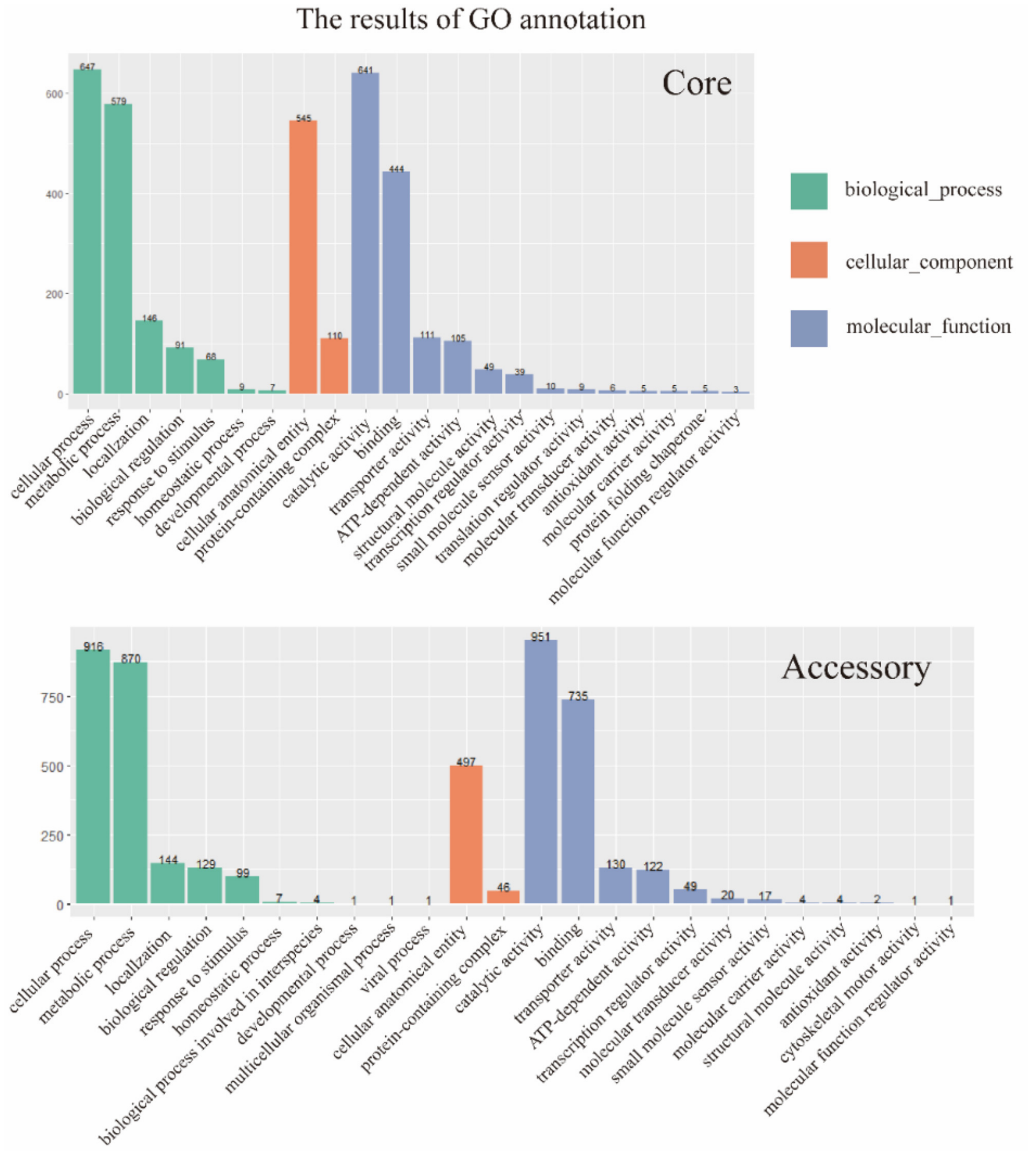
GO annotation of core genome and accessory genome. Despite variations in enrichment across biological processes, cellular components, and molecular functions, the core genome exhibited a higher number of annotations in cellular components, whereas the accessory genome showed significantly higher annotations in biological processes and molecular functions.

The two gene sets were aligned and annotated with the Interproscan (https://ftp.ebi.ac.uk/pub/software/unix/iprscan/, accessed on 25 September 2023), and the results were shown in the Figure 5. Compared to the accessory genome, although the core genome was widely involved in biological processes, molecular functions, and cellular components, the core genome had more annotations in the cellular component category. This reflected that the cellular structural positions where gene products execute functions were mainly encoded by the core genome. In addition, the accessory genome was also extensively involved in various processes that maintained bacterial survival, significantly enriching molecular function and biological process categories, showing certain essentiality, and possibly providing beneficial supplementation when the core genome was damaged.

Furthermore, the core genome and accessory genome had unique annotations in GO. Protein folding chaperone and translation regulator activities were unique GO annotations for the core genome, indicating the crucial role of the core genome in protein formation and regulation. The unique GO annotations for the accessory genome included biological processes involved in interspecies interactions, multicellular organism processes, viral processes, and cell skeleton movement activities. It revealed that the accessory genome might play a key role in resisting phage invasion, interacting with hosts, and providing a foundation for dynamic changes in cells.

### 3.7 The KEGG annotation results of the core genome and accessory genome

**Figure 6:**
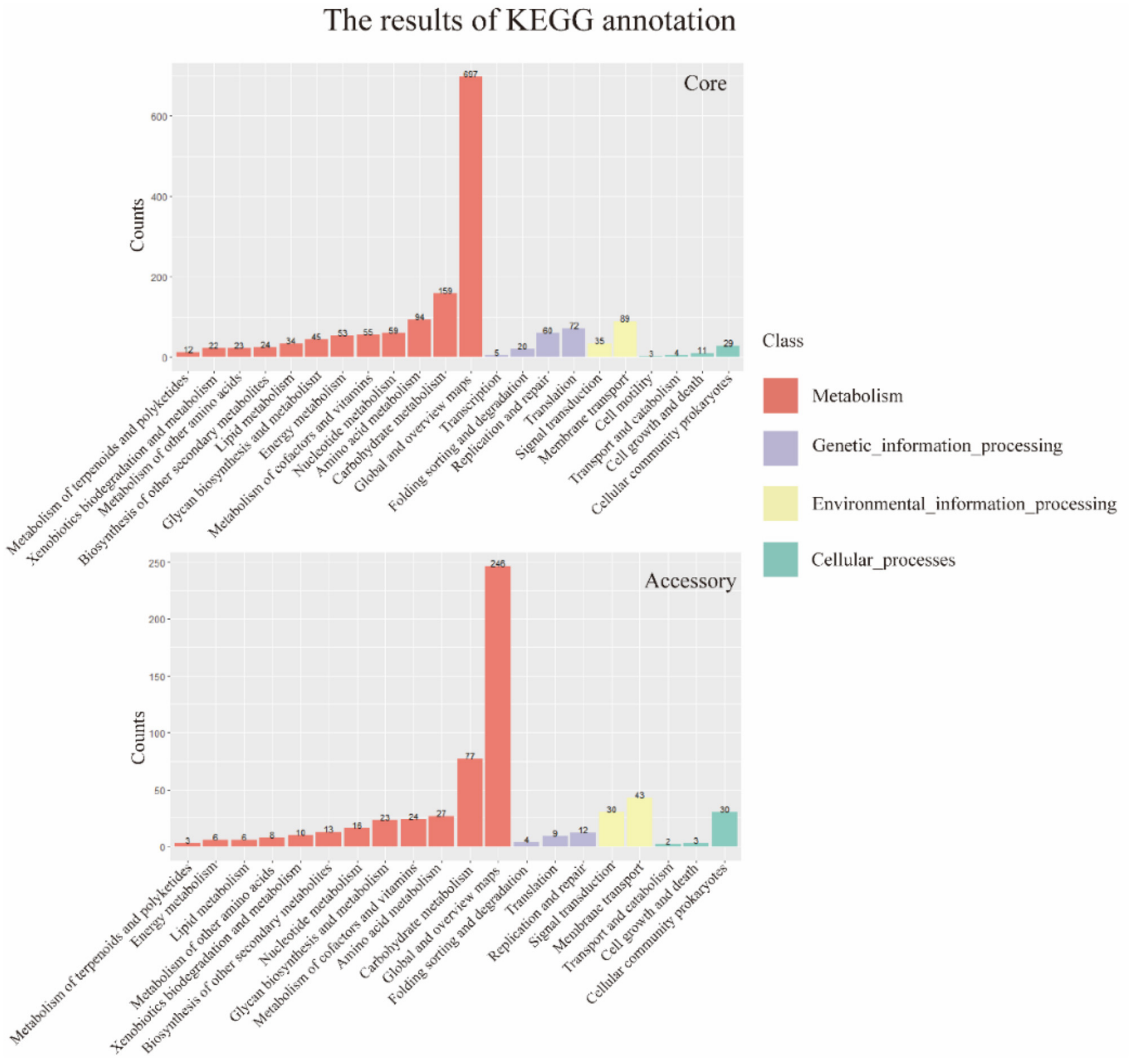
KEGG annotation of core genome and accessory genome. In general, the core genome demonstrates a significantly higher number of annotations in metabolism, genetic information processing, environmental information processing, and cellular processes compared to the accessory genome. Additionally, there are categories not covered by the accessory genome, such as transcription and cell motility.

The results of the alignment of the two gene sets with the Kyoto Encyclopedia of Genes and Genomes (KEGG, https://www.genome.jp/kegg/, accessed on 25 September 2023) database (Kanehisa and Goto, 2000) were shown in the Figure 6. The results indicated that the core genome was widely involved in the metabolism, genetic information processing, environmental information processing, and cellular processes of the bacterium. The annotation count was generally higher than that of the accessory genome. Moreover, it was annotated to pathways not covered by the accessory genome, such as transcription and cell motility, demonstrating the biological essentiality of the core genome. Similarly, the accessory genome also exhibited varying numbers of annotations in these four aspects, indicating a certain degree of biological essentiality, serving as a complement and modification to the core genome.

### 3.8 The VFDB annotation results of the core genome and accessory genome

**Figure 7:**
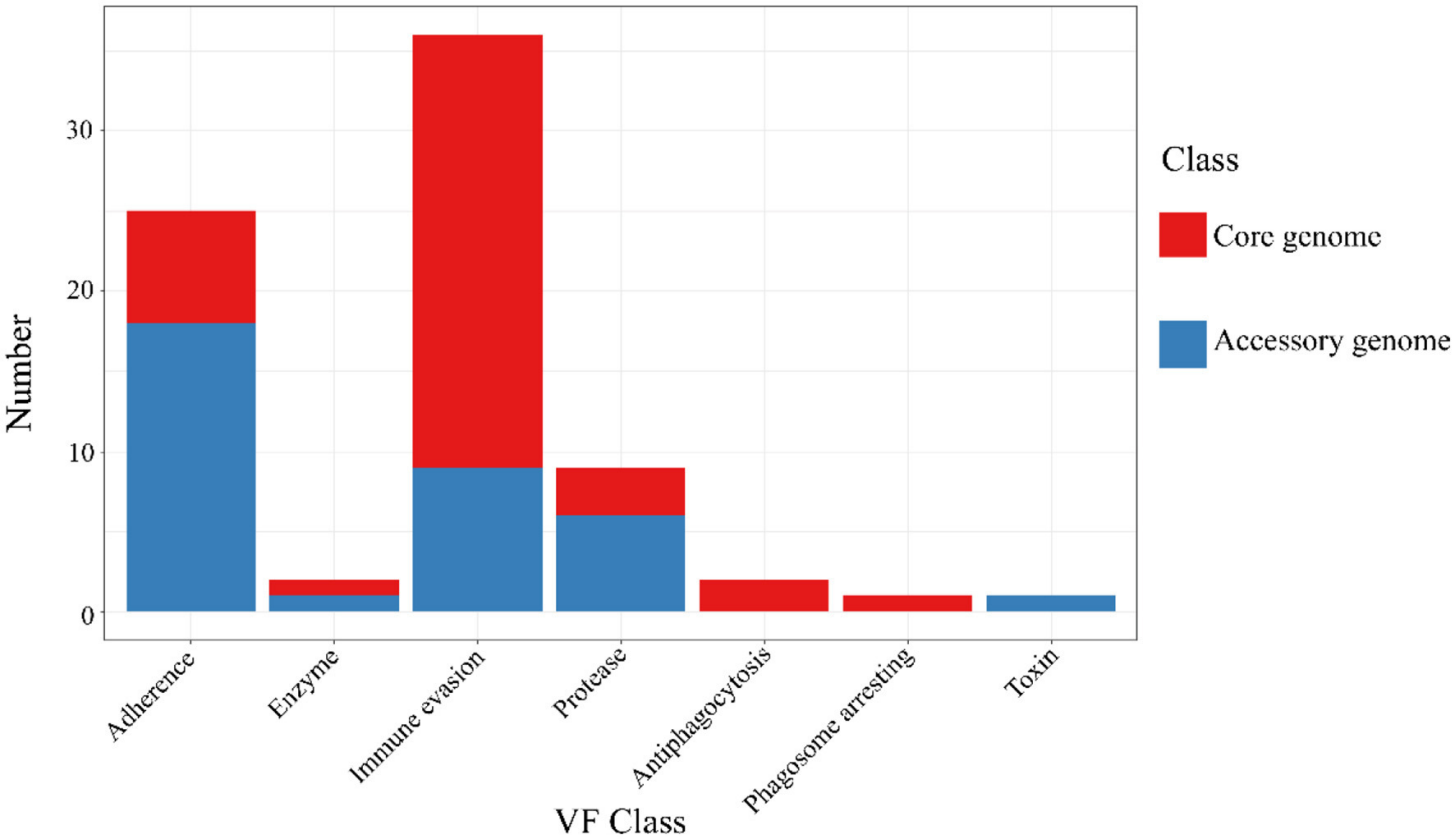
VFDB annotation of core genome and accessory genome. While both the core genome and accessory genome show varying degrees of annotations in adhesion, enzymes, immune invasion, and proteases, antiphagocytosis and phagosome arresting are unique annotation categories found in the core genome, whereas toxin is a unique annotation category in the accessory genome.

The results of the annotation of the two gene sets with the virulence factor database (VFDB, http://www.mgc.ac.cn/VFs/, accessed on 20 September 2023) database (Liu et al., 2022) to identify potential virulence factors. The results were shown below (Figure 7 and Table 6). The core genome and accessory genome obtained annotations for the following categories of virulence factors: adhesion, enzymes, immune invasion, proteases, anti-phagocytosis, phagosome capture, and toxins. Compared to the core genome, the accessory genome was completely lacking in the categories of anti-phagocytosis and phagosome capture, focusing primarily on adhesion and protease categories, and annotating virulence factors (toxins) not involved in the core genome.

**Table 6:** The virulence factor database (VFDB) annotation of the core genome and the accessory genome.

**VF class**	**Virulence factors**	**Related genes**	**Core genome**	**Accessory genome**
Adherence	Streptococcal lipoprotein rotamase A	*slrA*	1	NA
	Streptococcal plasmin receptor/GAPDH	*plr/gapA*	1	NA
	LrA islet	*srtC*	2	NA
	Fibronectin-binding proteins	*pavA*	1	NA
	Laminin-binding protein	*Lmb*	1	NA
	Sortase A	*srtA*	1	1
	Agglutinin receptor	Undetermined	NA	8
	Choline-binding proteins	*mrp*	NA	3
		*srtC4*	NA	2
		*srtB*	NA	1
		*cbpD*	NA	3
Enzyme	Streptococcal enolase	*eno*	1	NA
	Mitogenic factor 3	*mf3*	NA	1
Immune evasion	Polysaccharide capsule (Bacillus)	*manA*	1	NA
	Capsule	Undetermined	26	9
Protease	Serine protease	*htrA/degP*	1	NA
	Trigger factor	*tig/ropA*	1	NA
	Zinc metalloproteinase	*zmpC*	1	2
	C5a peptidase	*scpA/scpB*	NA	3
	Extracellular factor	*epf*	NA	1
Antiphagocytosis	Capsule (Enterococcus)	*cdsA*	1	NA
		*cps2j*	1	NA
Phagosome arresting	Nucleoside diphosphate kinase	*ndk*	1	NA
Toxin	Suilysin	*sly*	NA	1

Our findings indicated the presence of a diverse array of virulence factors in the pan-genome, potentially providing pathways for various pathogenic mechanisms of SS2. This diversity enhances the bacterium's survival opportunities and contributes to more severe pathological responses.

### 3.9 The CAZy annotation of results of the core genome and accessory genome

**Figure 8:**
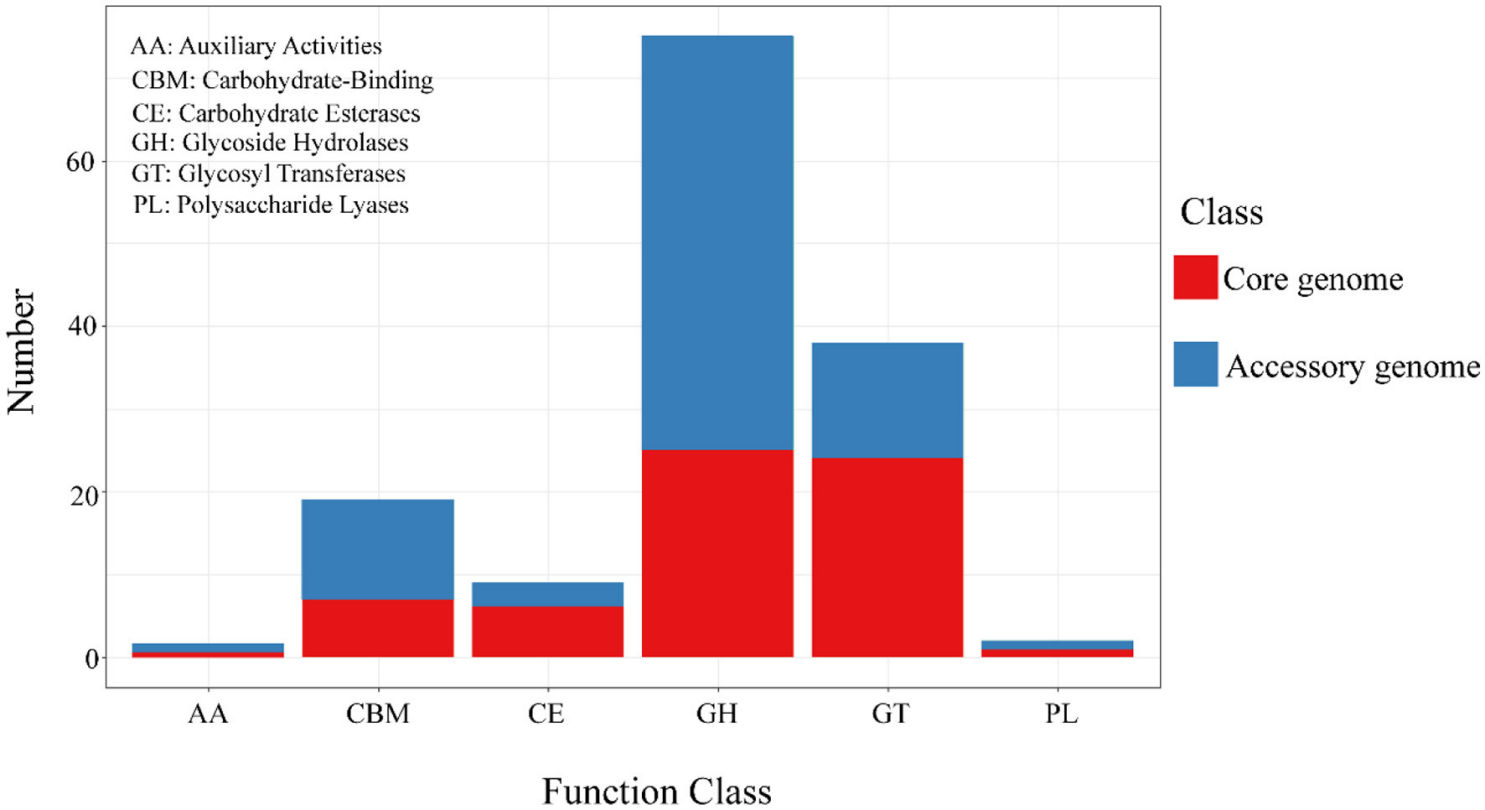
The results of CAZy annotation of core genome and accessory genome. Both the core genome and accessory genome exhibit varying numbers of annotations in all carbohydrate annotation categories, with a primary focus on GH and GT.

The alignment and annotation of the two gene sets with the Carbohydrate-Active enZYmes (CAZy, http://www.cazy.org/, accessed on 20 September 2023) database (Drula et al., 2022) were shown in the Figure 8 and Table 7. Both the core genome and accessory genome were involved in all categories, with the core genome primarily annotated as carbohydrate esterases (CE) and glycosyltransferases (GT). In contrast, the accessory genome was annotated with much more glycoside hydrolases (GH) and carbohydrate-binding modules (CBM) than the core genome.

**Table 7:** The Carbohydrate-Active enZYmes (CAZy) annotation of the core genome and the accessory genome.

**CAZy classification**	**Core genome**	**Accessory genome**
Auxiliary Activities (AA)	1	1
Carbohydrate-Binding Modules (CBM)	7	12
Carbohydrate Esterases (CE)	6	3
Glycoside Hydrolases (GH)	25	50
Glycosyl Transferases (GT)	24	14
Polysaccharide Lyases (PL)	1	1

### 3.10 The PHI annotation of the core genome and the accessory genome

**Figure 9:**
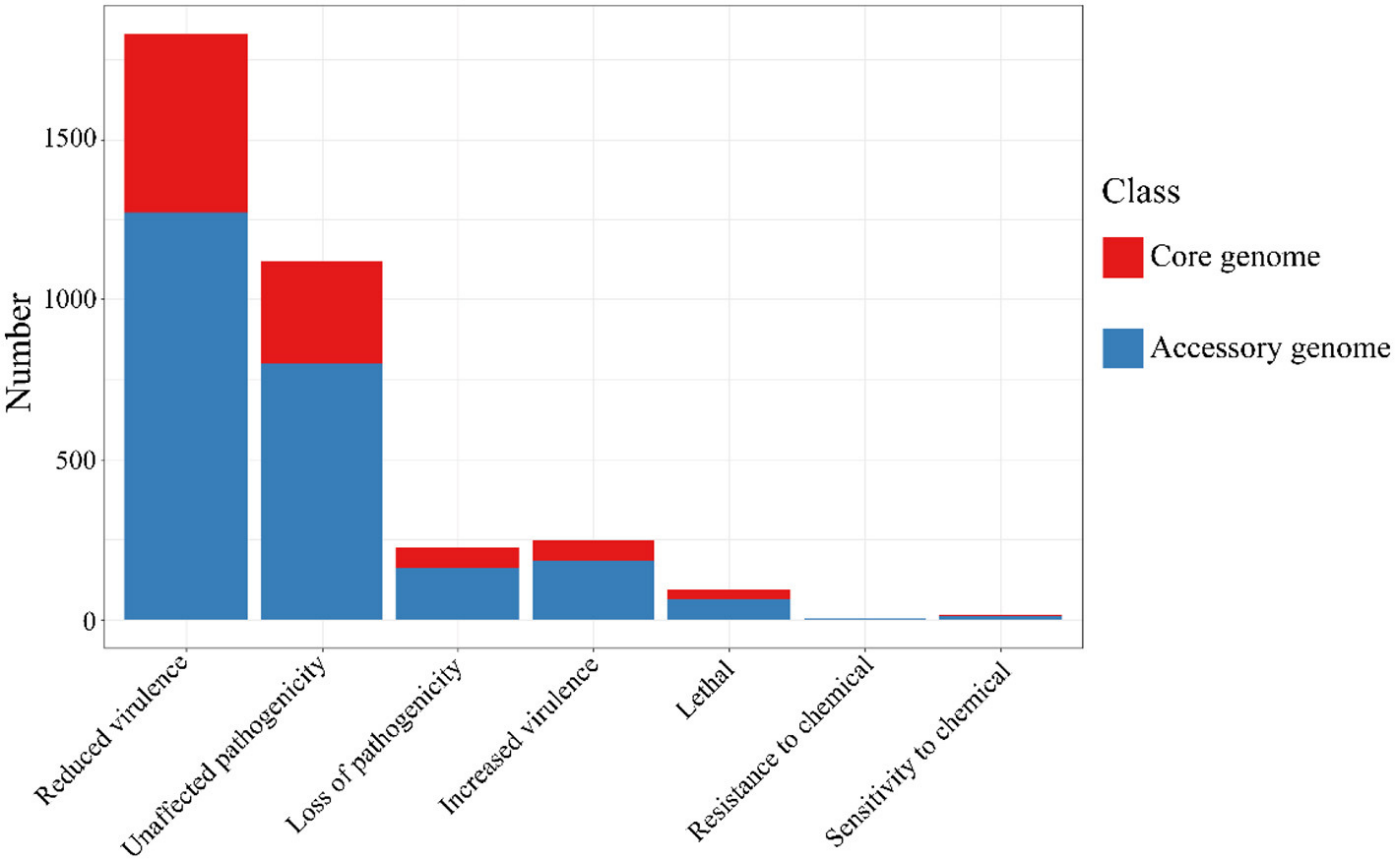
The results of PHI annotation of core genome and accessory genome. The accessory genome shows varying numbers of annotations in all PHI phenotype categories, including categories not covered by the core genome, such as resistance to chemical.

The alignment of the two gene sets with the Pathogen-Host Interaction (PHI, http://www.phi-base.org/, accessed on 20 September 2023) database (Urban et al., 2020) and subsequent annotation revealed that pathogenic genes were commonly present in both the core and accessory genomes (Figure 9 and Table 8). Furthermore, genes associated with different PHI phenotypes exhibited much higher abundance in the accessory genome compared to their abundance in the core genome. This observation was closely related to bacterial serum resistance, adhesion capabilities, and invasiveness.

**Table 8:** The Pathogen-Host Interactions (PHI) annotation of the core genome and the accessory genome.

**PHI phenotype classification**	**Core genome**	**Accessory genome**
Reduced virulence	560	1,271
Unaffected pathogenicity	321	797
Loss of pathogenicity	66	157
Increased virulence	66	183
Lethal	29	61
Resistance to chemical	1	2
Sensitivity to chemical	3	9

### 3.11 The CARD annotation of the cnzyss2-331 and pan-genome

**Figure 10:**
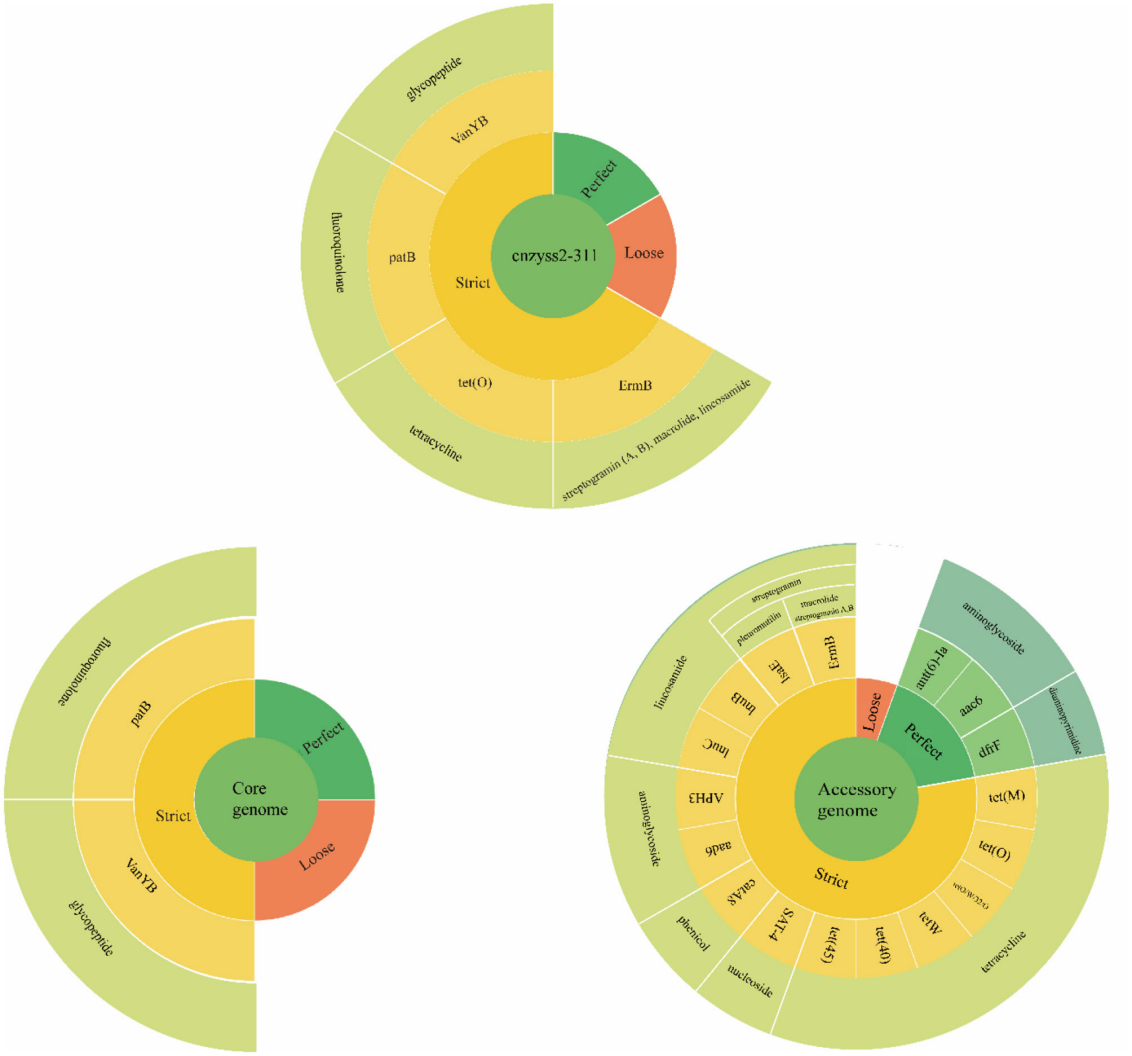
The results of CARD annotation of cnzyss2-311 and pan-genome. The figure provides a detailed depiction of the CARD annotation results for the isolated strains, core genome, and accessory genome. A large circle is composed of four categories, representing input data, matching patterns, AMR genes, and antibiotic classes from the innermost to the outermost layer. Additionally, white solid lines are used to separate different antibiotic classes.

The results of the annotation using RGI (V6.0.3, https://github.com/arpcard/rgi, accessed on 20 September 2023) against the comprehensive antibiotic resistance database (CARD, https://card.mcmaster.ca, accessed on 20 September 2023; Alcock et al., 2023) are shown in Figure 10. In the perfect or strict hits mode, four types of resistance genes were annotated in cnzyss2-331, namely *ErmB, tet(O), patB*, and *vanY* gene in *vanB* cluster, exhibiting resistance to streptogramins, macrolides, lincosamides, tetracyclines, fluoroquinolones, and glycopeptide antibiotics. These findings aligned well with the MIC test results in Table 4. Our results indicated that CARD annotation correlates with the antibiotic resistance phenotype.

The core genome and accessory genome were compared and annotated against the CARD database. The resistance mechanisms were categorized into six main types: antibiotic target alteration, antibiotic efflux, antibiotic inactivation, antibiotic target protection, antibiotic target replacement, and reduced antibiotic permeability (Figure 10 and Table 9). In the perfect or strict hits mode, the core genome was annotated with only two resistance genes, specifically, glycopeptide resistance genes and fluoroquinolone resistance genes. In contrast, the accessory genome contributed the majority of resistance genes (aminoglycosides, phenicol, streptogramin, macrolides, lincosamides, nucleosides, diaminopyrimidines, and tetracyclines), demonstrating a diverse array of resistance mechanisms, including antibiotic target alteration, antibiotic efflux, antibiotic inactivation, antibiotic target protection and antibiotic target replacement.

**Table 9:** The comprehensive antibiotic research database (CARD) annotation (perfect or strict hits).

**Class**	**Antibiotic resistance ontology (ARO) term**	**Drug class**	**Resistance mechanism**	**AMR gene family**
cnzyss2-311	*ErmB*	Streptogramin, macrolide, lincosamide, streptogramin B, and streptogramin A	Antibiotic target alteration	Erm 23S ribosomal RNA methyltransferase
	*tet(O)*	Tetracycline	Antibiotic target protection	Tetracycline-resistant ribosomal protection protein
	*patB*	Fluoroquinolone	Antibiotic efflux	ATP-binding cassette (ABC) antibiotic efflux pump
	*vanY gene in vanB cluster*	Glycopeptide	Antibiotic target alteration	Sugar-peptide resistance gene cluster
Core genome	*VanY* gene in vanB cluster	Glycopeptide	Antibiotic target alteration	Sugar-peptide resistance gene cluster
	*PatB*	Fluoroquinolone	Antibiotic efflux	ATP-binding cassette (ABC) antibiotic efflux pump
Accessory genome	*AAC6_30_AAC6_Ib*	Aminoglycoside	Antibiotic inactivation	Aminoglycoside bifunctional resistance protein
	*ANT(6)-Ia*	Aminoglycoside	Antibiotic inactivation	ANT(6)
	*aad(6)*	Aminoglycoside	Antibiotic inactivation	ANT(6)
	*APH(3′)-IIIa*	Aminoglycoside	Antibiotic inactivation	APH(3′)
	*catA8*	Phenicol	Antibiotic inactivation	Chloramphenicol acetyltransferase (CAT)
	*ErmB*	Streptogramin, macrolide, lincosamide, streptogramin B, and streptogramin A	Antibiotic target alteration	Erm 23S ribosomal RNA methyltransferase
	*LnuC*	Lincosamide	Antibiotic inactivation	Lincosamide nucleotidyltransferase (LNU)
	*LnuB*	Lincosamide	Antibiotic inactivation	Lincosamide nucleotidyltransferase (LNU)
	*LsaE*	Lincosamide, pleuromutilin, and streptogramin	Antibiotic target protection	Lsa-type ABC-F protein
	*SAT-4*	Nucleoside	Antibiotic inactivation	Streptothricin acetyltransferase (SAT)
	*DfrF*	Diaminopyrimidine	Antibiotic target replacement	Trimethoprim resistant dihydrofolate reductase dfr
	*Tet45*	Tetracycline	Antibiotic efflux	MFS antibiotic efflux pump
	*Tet40*	Tetracycline	Antibiotic efflux	MFS antibiotic efflux pump
	*TetM*	Tetracycline	Antibiotic target protection	Tetracycline-resistant ribosomal protection protein
	*TetW*	Tetracycline	Antibiotic target protection	Tetracycline-resistant ribosomal protection protein
	*TetO*	Tetracycline	Antibiotic target protection	Tetracycline-resistant ribosomal protection protein
	*tet(O/W/32/O)*	Tetracycline	Antibiotic target protection	Tetracycline-resistant ribosomal protection protein

## 4 Discussion

Pan-genome analysis provides a comprehensive understanding of the overall lineage of SS2. It can be used to screen and identify various virulence and antibiotic resistance genes. In this study, the strain cnzyss2-311 was initially assembled into a genomic sketch using third-generation sequencing data, and second-generation sequencing data were used for error correction, resulting in the completion of the full genomic sequence. Subsequently, pan-genome analysis of SS2 revealed that only 1353 genes were shared among different individuals, constituting the core genome (Figure 3). The core genome serves as the essential framework supporting the rest of the genome, rather than the minimal set of genes required for bacterial survival (Medini et al., 2005; Tettelin et al., 2008). If we expand the definition of the core (including genes that are only partially missing in a small fraction of the genomes), the core genome consists of 1,458 genes, including both core and soft genes. These genes are present in at least 95% of the sampled genomes. The results suggested that SS2 has an open pan-genome, and the number of core genome genes does not significantly change with an increasing number of strains and consistent with previous research (Guo et al., 2021). In contrast, genes present in certain strains and those unique to individual strains constitute the accessory genome, comprising 4,337 genes. The accessory genome reflects the genetic diversity and unique genomic features present in specific strains (Kim et al., 2020). Pan-genome analysis provides insights into the genomic variability of SS2, highlighting both the conserved core genes and the flexible accessory genes that contribute to its adaptability and diversity across strains.

The core genome and accessory genome are extensively involved in various aspects of bacterial life activities, including metabolism and genetic variation (Figures 4–6). The core genome primarily focuses on the transport, metabolism, and translation processes of essential substances for life. Similarly, the accessory genome exhibits certain biological characteristics, especially in functions such as carbohydrate transport and metabolism, transcription, etc. These examples indicate that the primary function of the core genome is to control the normal morphology, reproduction, and execution of basic biological functions in bacteria. In addition, genes participating in fundamental biological processes have been discovered in the accessory genome. It is speculated that when the core genome is damaged, the accessory genome may have alternative functions to maintain certain biological processes in bacteria.

Similarly, the core genome and accessory genome exhibit differences in the expression of carbohydrate enzymes (Figure 8). For example, the annotation of the core genome revealed a high abundance of CE and GT. These are associated with the hydrolysis of carbohydrates and the formation of glycosidic bonds (Venegas et al., 2022). It plays a crucial role in bacteria's acquisition of external carbon sources, adhesion to host cells, and biofilm formation (Middleton et al., 2021; Na et al., 2021). In addition, the accessory genome exhibits significantly higher levels of GH compared to the core genome. GH is mainly involved in the hydrolysis of polysaccharide glycosidic bonds, enabling the degradation of complex host polysaccharides, facilitating bacterial acquisition of carbohydrate nutrients, and promoting the colonization of *Streptococcus suis* (Chen et al., 2020; Hamre and Sørlie, 2020; Redman et al., 2020).

In general, bacterial adhesion to the surface of objects to form a biofilm is crucial for further infecting the host (Fittipaldi et al., 2012). SS2 typically colonizes the upper respiratory tract, and crossing the mucosal barrier is a prerequisite for SS2 to cause invasive infections (Xia et al., 2019). The annotation results of virulence factors also suggest that, aside from undefined capsule-related genes, the most abundantly annotated virulence genes in the pan-genome belong to the adhesion category. Streptococcal lipoteichoic acid rotamase A (SlrA) has been shown to indirectly promote host cell adhesion and invasion, as well as prevent phagocytosis of *Streptococcus pneumoniae*, making it a potential therapeutic target for preventing bacterial colonization (Cron et al., 2009). Additionally, SS2 possesses a homologous lipoteichoic acid protein gene (*slrA*), but lacks choline-binding proteins (CBPs; Hermans et al., 2006). Interestingly, the virulence factor annotation results also show that CBPs are completely absent in the core genome of SS2. CBPs have been shown to play a crucial role in the growth, autolysis, and biofilm formation of *S. pneumoniae* and are essential for its pathogenicity (Galán-Bartual et al., 2015). Moreover, in Gram-positive bacteria, various surface proteins decorated with proteins are accomplished by sortase (Spirig et al., 2011). Although not essential for bacterial viability, sortase may be an important virulence factor, as it is involved in mediating bacterial adhesion to host tissues, host cell entry, evasion and inhibition of the immune response, and acquisition of essential nutrients by surface proteins (Marraffini et al., 2006). Our results show that streptococcal sortase A (*srtA*) and pilin-sorting enzyme C (*srtC*) are fixed encoded sortases in SS2. SrtA recognizes specific LPXTG motifs, anchoring a diverse array of functionally distinct proteins to the cell wall, playing a crucial role in adhesion to extracellular matrix (ECM) components (Spirig et al., 2011; Li et al., 2013; Alharthi et al., 2021). Considering the essential role of SrtA in the pathogenic mechanisms of Gram-positive bacteria, it is also considered an ideal target for potential drugs (Spirig et al., 2011). SrtC sorting enzyme not only anchors smaller substrates but also participates in pilus assembly, suggesting that the formation of pili is an inherent ability of SS2 to promote microbial adhesion and biofilm formation (LeMieux et al., 2008; Qian et al., 2018; Faulds-Pain et al., 2019). In addition, during the process of adhering to endothelial cells and epithelial cells in host tissues, SS2 mediates host cell invasion through its own ECM-binding protein, binding to host ECM components fibronectin (FN) and laminin (LN; Schwarz-Linek et al., 2003; Wahid et al., 2005; Ragunathan et al., 2009). Interestingly, highly conserved hexapeptide sequences (LPXTGE) have been identified in the C-terminus of known surface proteins in Gram-positive cocci and play an important role in cell adhesion, such as the aforementioned sortases, fibronectin-binding proteins (FnBPs), and laminin-binding proteins (LNBPs; Fischetti et al., 1990; Tenenbaum et al., 2007; Yamaguchi et al., 2013). However, there is also research indicating that surface proteins encoded by the *pavA* gene, which lacks a typical LPXTG anchoring motif, still play an important role in the adhesion and virulence of *Streptococcus pneumoniae* (Pracht et al., 2005). In conclusion, the core genome of SS2 contains diverse adhesion factors and adhesion proteins, and surface proteins with LPXTG anchoring motifs may not be the sole criterion driving the interaction between the bacterium and the host.

Interestingly, in the core genome, enolase (eno) was identified as an essential virulence factor for *Streptococcus suis* serotype 2 (Xia et al., 2019). It imparts the bacterium with the ability to directly produce toxicity to porcine brain microvascular endothelial cells (PBMECs), promoting cell apoptosis, inhibiting the expression of tight junctions, or activating the ERK and p38MAPK signaling pathways in porcine brain microvascular endothelial cells to secrete the pro-inflammatory factor IL-8, and increasing the permeability of the blood-brain barrier (Liu H. et al., 2021). Therefore, even in strains considered non-virulent or low-virulent, there is still a possibility of penetrating the blood-brain barrier, which may be one of the reasons for SS2 causing host meningitis. Moreover, classical virulence factors of *Streptococcus suis*, such as sly, muramidase-released protein (mrp), and extracellular factor (ef), are found exclusively in the accessory genome (Li et al., 2017). For example, suilysin (sly), as one of the cholesterol-dependent bacterial cytolysins, exhibits direct cytotoxicity (Vötsch et al., 2019). Sly induces cell membrane rupture, decreased cytoplasm density, and even exudation through perforation, leading to lesions and damage to epithelial cells and fibroblasts from different host sources (Liu M. et al., 2021). It is a recognized important virulence factor of SS2. This phenomenon suggests that these virulence factors could serve as criteria for assessing the virulence of SS2, as their presence varies among different strains, as previously reported.

In addition, only in the core genome, virulence genes related to anti-phagocytosis and phagosome capture, such as *cps2J, cdsA*, and *ndk*, were found. Cps is a recognized virulence factor, not only an important basis for the serotype classification of *Streptococcus suis*, but also an important factor hindering phagocytosis. Its presence requires the combined action of immunoglobulins and complement to promote phagocytosis (Zhao et al., 2015). In addition, researchers have found that the capsule switch in *Streptococcus suis* can be achieved through gradual evolution with a combination of minor mutation, deletion, and recombination in the cps locus (Zhu et al., 2020). Therefore, this may also be the reason why *cps* genes were found in both the core genome and accessory genome. Phosphatidylglycerol transferase, encoded by *cdsA* gene, is a major enzyme in the synthesis of cell membrane phospholipids (Adams et al., 2017; Sawasato et al., 2019). Although studies have found that it mediates resistance to daptomycin in streptococci and enterococci and may resist innate and exogenous antimicrobial peptide attacks, the specific anti-phagocytic mechanism remains unclear (Mishra et al., 2017; Tran et al., 2019). In addition, nucleoside diphosphate kinase (ndk) is a nucleotide metabolism enzyme that not only maintains the ribonucleotide and deoxyribonucleotide pools in cells but also participates in the regulation of virulence-related traits related to quorum sensing systems (QS), Type III Secretion Systems (T3SS), and virulence factor production systems in *Pseudomonas aeruginosa* (Neeld et al., 2014; Yu et al., 2016, 2017). Moreover, the ndk in *Mycobacterium tuberculosis* has macrophage toxicity and plays a crucial role in evading the host immune system's elimination (Chopra et al., 2003). In summary, the core genome serves as the fundamental assurance for the bacterium to invade the host, while the virulence factors present in the accessory genome complement the core genome advantageously, providing various pathways for bacterial adhesion to the host and evasion of the immune system. Moreover, various virulence factors existing in the core genome indicated that strains previously considered non-virulent may still contribute to infection. The diverse virulence factors present in the accessory genome can be viewed as an effective complement to the core genome, influencing the pathogenicity of the bacterium. The redundancy of various virulence factors in *Streptococcus suis* allows them to compensate for the loss of another factor. Finally, non-virulent strains also have the possibility of acquiring virulence genes and transforming into virulent strains (Griffith, 1928; Tribble et al., 2012).

It is generally believed that antimicrobial resistance can be categorized into intrinsic resistance and acquired resistance based on its underlying causes (Olaitan et al., 2014; Zhang and Feng, 2016). While acquired resistance is commonly considered the major factor leading to the development of resistant bacteria, intrinsic resistance may still pose challenges to the treatment of bacterial infections (Sirichoat et al., 2020). According to the annotation results from the CARD database, the core genome of SS2 contains two types of antibiotic resistance genes, namely *patB* and *vanY* (Figure 10 and Table 9). *PatB* is a natural resistance gene in *Streptococcus pneumoniae*, conferring resistance to fluoroquinolone antibiotics through the ABC family (El Garch et al., 2010). This result supported previous research, indicating that SS2 achieves efflux of fluoroquinolones through SatAB (an ABC transporter homologous to PatA and PatB; Escudero et al., 2011). Gram-positive bacteria like lactobacilli have natural resistance to glycopeptide antibiotics (such as vancomycin) controlled by the *vanY* gene (Finch et al., 2010; Alby and Miller, 2018). The results suggest that SS2 may possess natural resistance to fluoroquinolone and glycopeptide antibiotics. Remarkably, despite the single strain showing sensitivity to ofloxacin (S, fluoroquinolones) based on MIC results, it demonstrated resistance to levofloxacin (I, fluoroquinolones), trovafloxacin (S, fluoroquinolones), and vancomycin (S, Glycopeptides; Table 4). The distinct phenotypes of the strain toward these three fluoroquinolones suggest that the resistance mechanism mediated by *patB* may not uniformly affect antibiotics of the same class.

Additionally, bacteria can acquire resistance to a particular class of antibiotics through various mechanisms such as mutation, transformation, and integration of exogenous DNA (Magiorakos et al., 2012; Impey et al., 2020). The CARD annotation for the single strain identified only four classes of resistance genes, and the represented resistant drugs align well with the MIC results. When compared to the CARD annotation results for the individual strain cnzyss2-311, the pan-genome shows a more diverse overall resistance profile with a greater variety of resistance mechanisms (Figure 10). Notably, the tetracycline resistance gene *tet* was carried at high frequencies, consistent with previous reports (Haenni et al., 2018). Therefore, the accessory genome is a major contributor to bacterial acquisition of antibiotic resistance. Our results indicate a close correlation between antibiotic resistance phenotypes and the composition of CARD-related genes, highlighting the accessory genome as a primary source of antibiotic resistance. The acquisition and loss of resistance genes contribute to the challenges in clinical treatment. Antibiotic therapy is currently a major approach for managing *Streptococcus suis* infections in pigs, and this may lead to the widespread dissemination of antimicrobial resistance (AMR) genes, adding pressure to clinical treatment. Thus, it is essential to regulate antibiotic use in clinical practice. These annotation results indicate that SS2's main reservoir of resistance genes comes from the accessory genome. Bacteria can acquire exogenous resistance genes through various means, and the continuous acquisition or loss of resistance genes plays a crucial role in adapting to environmental pressures. This phenomenon is likely a direct cause of the current challenges in clinical treatment.

In this study, various databases and annotation tools were employed, as shown in Table 1. Specifically, eggnog-mapper was used for alignment with the COG database, providing annotation results including COG annotations, GO annotations, and KEGG annotations. Considering that the GO descriptions in the COG database might not be the latest version, we conducted a reannotation using the Interproscan database. Additionally, we excluded some descriptions unrelated to bacteria in the KEGG annotations, such as human cancers, to ensure the accuracy of KEGG annotations.

Our analysis has some limitations. Firstly, we are confined to publicly available genomes retrieved from NCBI, including a total of 229 isolates, and the genomes assembled in this study. Lastly, as our analysis is limited to assumed proteins in the database, further experiments are required to validate these presumed genes associated with virulence and drug resistance.

In summary, we have demonstrated the characteristic differences between the core genome and accessory genome of SS2. Additionally, we have identified potential virulence genes associated with SS2, laying the foundation for the exploration of new virulence factors. During the early stages of infection, multiple genes influence the adhesion of the bacterium and may contribute to the pathogenicity of SS2. The presence of diverse virulence factors in SS2 suggests redundancy, possibly serving as advantageous supplements to other virulence factors. Finally, our findings indicate that SS2 may exhibit natural resistance to fluoroquinolone and glycopeptide antibiotics.

## Data availability statement

The datasets presented in this study can be found in online repositories. The names of the repository/repositories and accession number(s) can be found in the article/Supplementary material.

## Author contributions

YZ: Writing – original draft, Writing – review & editing. TT: Conceptualization, Data curation, Formal analysis, Funding acquisition, Investigation, Methodology, Project administration, Resources, Software, Supervision, Validation, Visualization, Writing – review & editing. XY: Data curation, Project administration, Resources, Supervision, Writing – review & editing. YL: Data curation, Investigation, Project administration, Writing – review & editing. ZY: Conceptualization, Data curation, Investigation, Visualization, Formal analysis, Funding acquisition, Methodology, Project administration, Resources, Software, Supervision, Validation, Writing – review & editing. MR: Data curation, Formal analysis, Supervision, Software, Validation, Visualization, Writing – review & editing. GZ: Conceptualization, Software, Visualization, Methodology, Supervision, Validation, Writing – review & editing. YY: Project administration, Funding acquisition, Visualization, Writing – review & editing. AL: Formal analysis, Resources, Supervision, Validation, Visualization, Writing – review & editing. YW: Conceptualization, Funding acquisition, Validation, Writing – original draft, Writing – review & editing.
